# Trajectory of irritability in children and youth in Ontario, Canada, throughout the COVID‐19 pandemic

**DOI:** 10.1002/jcv2.70119

**Published:** 2026-05-06

**Authors:** Theodore C. K. Cheung, Katherine T. Cost, Ronda F. Lo, Evdokia Anagnostou, Catherine S. Birken, Alice Charach, Suneeta Monga, Elizabeth Kelley, Rob Nicolson, Paul D. Arnold, Christie Burton, Daphne J. Korczak, Jennifer Crosbie

**Affiliations:** ^1^ Department of Psychiatry The Hospital for Sick Children Toronto Ontario Canada; ^2^ Neurosciences and Mental Health SickKids Research Institute The Hospital for Sick Children Toronto Ontario Canada; ^3^ Department of Psychology University of Waterloo Waterloo Ontario Canada; ^4^ Department of Psychology Toronto Metropolitan University Toronto Ontario Canada; ^5^ Department of Pediatrics Temerty Faculty of Medicine University of Toronto Toronto Ontario Canada; ^6^ Department of Pediatrics Holland Bloorview Kids Rehabilitation Hospital Toronto Ontario Canada; ^7^ Division of Paediatric Medicine The Hospital for Sick Children Toronto Ontario Canada; ^8^ Department of Psychiatry Temerty Faculty of Medicine University of Toronto Toronto Ontario Canada; ^9^ Departments of Psychology and Psychiatry Queens University Kingston Ontario Canada; ^10^ Department of Psychiatry University of Western Ontario London Ontario Canada; ^11^ Mathison Centre for Mental Health Research and Education Hotchkiss Brain Institute University of Calgary Calgary Alberta Canada; ^12^ Department of Psychiatry Cumming School of Medicine University of Calgary Calgary Alberta Canada

**Keywords:** child and adolescent, COVID‐19, irritability, longitudinal study, resilience

## Abstract

**Background:**

Children and youth have been greatly affected by the COVID‐19 pandemic. The current study explored trajectories of irritability across the pandemic and associations with mental health outcomes. Models included risk and protective factors; age, gender, ethnicity, family income, pre‐existing mental health condition, parental mental health, and resilience.

**Methods:**

The study included 905 children and youth (mean age: 11.85, SD: 2.92, range: 8–17 years old: male: 55%) from the longitudinal Ontario COVID‐19 and Kids Mental health Study. Irritability was measured based on parent and self‐reports over seven time points from April 2020 to June 2022. Latent growth curve modeling captured the initial level of irritability with the intercept and change over time with the slope as the latent factors, which were in turn used to predict internalizing and externalizing mental health outcomes using multiple regression.

**Results:**

Model fits for both parent‐report and self‐report data were satisfactory, showing an initial increase (only in parent‐report data) and a linear decrease overtime. In both models, having a younger age, female gender (only in self‐report), higher parental anxiety, positive mental health history and lower resilience were significantly associated with higher initial irritability level (*p*s < 0.001; but not ethnicity and family income, *p*s > 0.05), while only younger age associated with the steeper drop in irritability over time (*p* = 0.005). Regression analysis showed that a greater decrease in irritability symptoms over time was associated with lower depression, anxiety, inattention and hyperactivity/impulsivity symptom levels at the end of the study period, with *F*s > 54.59, *p*s < 0.001.

**Conclusion:**

The results showed that pediatric irritability levels were sensitive to the COVID‐19 pandemic, and generally decreased over time. Risk and protective factors mostly affected the initial level while age was associated with the change of irritability over time. Early signs of irritability, especially among those high‐risk groups, could potentially inform patterns of mental health outcomes in the face of adversity as a potential target for early intervention for clinicians and educators.

## Key Points

**What's known:**

- The COVID‐19 pandemic has had a profound impact on the mental health of children and adolescents, with limited research focusing on irritability as a transdiagnostic and sensitive indicator of psychological distress.

**What's new:**

- This study identifies key predictors of irritability trajectories during the pandemic, including age, parental anxiety, resilience, and pre‐existing mental health conditions.
- Using a trajectory approach, it was found that greater reductions in irritability over time were associated with lower levels of depressive symptoms, anxiety, inattention, and hyperactivity/impulsivity.

**What's relevant:**

- Irritability emerges as a marker for identifying children and adolescents at elevated risk for adverse mental health outcomes, offering valuable insights for early intervention strategies in future public health emergencies.

## Graphical abstract

This study explored how irritability in children and youth changed during the COVID‐19 pandemic and its impact on mental health. Results showed a gradual decline in irritability, with factors like age, parental anxiety, and resilience influencing its trajectory. A greater reduction was linked to better mental health outcomes, highlighting irritability as a key target for early intervention.

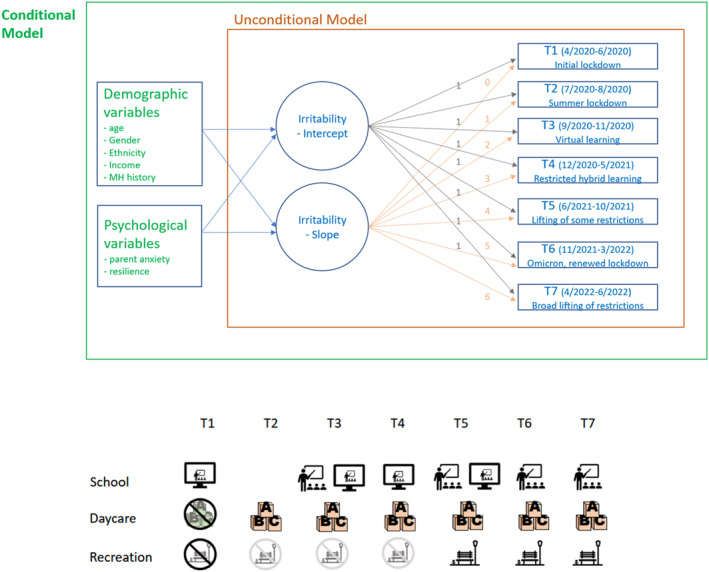

## INTRODUCTION

Many studies have been published on understanding the impacts of the COVID‐19 pandemic as a global public health crisis. While early studies were focused on understanding the pathology (Condie, 2020; Husain, 2021; Mallapaty, 2021) and potential treatments (Dagan et al., 2021; Polack et al., 2020; Rubin & Longo, 2020), later studies addressed the mental health impacts on children (Bignardi et al., 2021; Fong & Iarocci, 2021; Korczak et al., 2022; Nikolaidis et al., 2021a; Racine et al., 2020; Samji et al., 2022; Vaillancourt et al., 2021; for review, see Madigan et al., 2023) and adults (Cysique et al., 2022; Czeisler et al., 2020; Danielsen et al., 2023; Krygsman et al., 2023; Nikolaidis et al., 2021a). Although the World Health Organization (WHO) officially announced the end of the pandemic (as a public health emergency) in May 2023, the pandemic has provided an unprecedented opportunity to understand how children cope with massive changes in life routines alongside the perceived threat that lasted for over two years (Chen et al., 2022; Cost et al., 2021a; Dvorsky et al., 2021; Fruehwirth et al., 2021; Korczak et al., 2024; Nikolaidis et al., 2021a). Namely, studies found increased depression symptoms, but fewer changes in anxiety and posttraumatic stress symptoms in children (Bignardi et al., 2021; Chen et al., 2022). In addition, various individual factors such as worry, optimism, fear of COVID, diagnosis of ADHD, and coping flexibility; and contextual factors like life events, community satisfaction, and family support have been found to mediate mental health outcomes (Chen et al., 2022; Dvorsky et al., 2021a; Pearman et al., 2021; Rizeq et al., 2021). Few studies, however, have examined irritability in children and youth in the context of the COVID‐19 pandemic (Dönmez & Uçur, 2021). Irritability is an important mental health construct given that it is transdiagnostic (Kishida et al., 2022). It is thus not too surprising that, during the pandemic, irritability symptoms were found even more common (51.4%) among children than depressive features (29.5% (Dönmez & Uçur, 2021). Tracking the changes of irritability not only provides insight into the long‐term consequences of the pandemic on children's mental health at the group level, but it also elucidates how the pattern of changes may predict individual differences in mental health outcomes (Galatzer‐Levy et al., 2018; McArdle & Epstein, 1987).

In the face of the COVID‐19 pandemic, children reacted differently based on different demographic, individual (e.g., gender, family income, mental health history) and psychological (e.g., resilience, coping strategies, social connectedness, and parental mental health) factors (Madigan et al., 2023; Masten et al., 2021; Racine et al., 2020; Samji et al., 2022; Taylor, 2022a). In a review by Samji et al. (2022), it was found that among children and adolescents, poorer mental health outcomes were associated with older age, female gender, neurodivergence, and presence of chronic physical and mental conditions during the first year of the COVID‐19 pandemic (data up to February 2021). In addition, research showed that in the face of adverse events, resilience (Bonanno et al., 2011, 2023; Galatzer‐Levy et al., 2018) could be a promotive and protective variable, despite other pre‐existing risk factors that could put children in high risk for poor coping and susceptibility to onset of psychopathology (Masten et al., 2021; Samji et al., 2022). Belonging to Racial/ethnic minoritized group, and having mid to high family incomes, had slight to small effect in worsening of anxiety symptoms (cf. Madigan et al., 2023). Understanding how demographic and psychological factors contribute to trajectories of irritability, rather than only irritability level at baseline per se, can contribute to discovering effective mitigation strategies and help practitioners and policymakers attend to high‐risk children and youths to help mitigate the impacts on mental health earlier (Bonanno et al., 2023; Yuan, 2021a).

The current study used a trajectory‐based approach (Galatzer‐Levy et al., 2018) to examine the variation in irritability symptoms across the COVID‐19 pandemic as a function of demographic (age group, gender, ethnicity, family income, and mental health history) and psychological (parental anxiety level, resilience) variables. Lastly, we tested whether the initial and the change of irritability associated with lower internalizing (depression and anxiety symptoms) and externalizing (ADHD symptoms) outcomes.

## METHOD

### Public health context during the study timeframe

**FIGURE 1:**
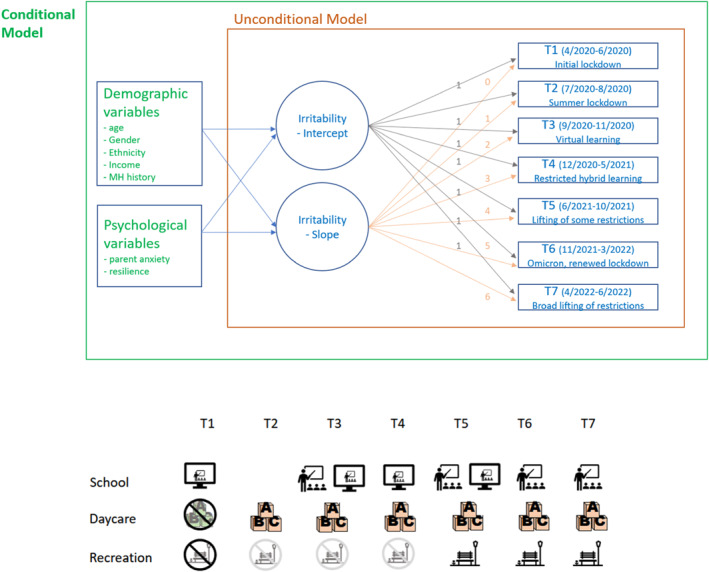
The conceptual model. Unconditional model: the trajectory of irritability was depicted by the seven time points with underlying intercept and slope within the LGCM. Conditional model: demographic variables and psychological variables were included in the LGCM. Regression model: The intercepts and slopes from the conditional model were independently used as regressors to predict mental health outcomes. School: virtual/in‐person/hybrid; Daycare: present/absent; Recreation: closed/partial/re‐opened. LGCM, latent growth curve model.

The current study captured the time periods from April 2020 to June 2022, which covered the acute phase in reaction to the first wave of COVID‐19 pandemic, the different waves and lockdowns in Ontario, to the re‐opening phase as marked by the end of the mask mandate (see Table S1 for details). Ontario experienced the first wave of the pandemic from March to June 2020. During this first time period (T1), all public and private facilities were closed (see Table S1 for the descriptions of key events from T1 to T7; Figure 1 for the graphical overview). By the end of the October 2021 (T5), about 40% of Ontario population were fully vaccinated (two doses). The last time period (T7) covered the Ontario reopening plan from March to the end of June 2022, when the face mask mandate was removed. During this period, about 80% of Ontario adult population, 42% of children (age 5–11), and 84% of adolescents (age 12–17) were fully vaccinated.

### Participants and procedures

Data for this study were collected as part of the Ontario COVID‐19 and Kids Mental Health study (Korczak et al., 2022), a collaboration of four cohorts in Ontario, Canada, each with an existing participant base, to examine the effects of the COVID‐19 pandemic on children, youth and families. Detail regarding individual cohorts and the overall study procedure has been previously described by (Korczak et al., 2022). Briefly, the collaboration is comprised of two clinically‐referred mental health and neurodevelopmental (NDDs) cohorts and two community cohorts: (i) SickKids Psychiatry cohort: children/adolescents referred to an outpatient MH clinic for evaluation of MH concerns including but not limited to depression and anxiety disorders, attention‐deficit/hyperactivity disorder (ADHD), obsessive‐compulsive disorder (OCD), disruptive behavior disorders; (ii) the Province of Ontario Neurodevelopmental Disorder (POND) Network: children/adolescents in the community with neurodevelopmental disorders (NDDs), including autism, ADHD, OCD, and intellectual disability (POND Network, 2023); (iii) The Applied Research Group for Kids (TARGet Kids!): healthy children recruited from birth to age 7 years in the Greater Toronto Area and participating in a primary care practice‐based research network (Carsley et al., 2015); and (iv) Spit for Science, a population‐based sample of children/adolescents recruited at an urban science museum (Burton et al., 2019; Spit for Science, 2023). Three cohorts from above (except iii) were selected that include 8–18 years old children and adolescents with and without pre‐existing mental health and NDDs (see Table 1 for demographic information).

**TABLE 1:** Demographic Information and covariate measures.

	Parent report (8–18 years)	Child (self) report (10–18 years)
	*n* = 916	*n* = 501
Child age—Mean (SD)	11.81 (2.95)	13.14 (2.48)
Young (<13 years old)	62.7% (574)	43.9% (220)
Old (≥13 years old)	37.3% (342)	56.1% (281)
Gender of child—% (*n*)		
Male	55.5% (508)	48.9% (245)
Female	43.3% (397)	46.5% (233)
Other/did not respond	1.2% (11)	4.6% (23)
Parent‐report family income—% (*n*)		
Less than $80,000	23.6% (216)	21.2% (106)
Over $80,000	60.9% (558)	58.1% (291)
Did not respond	15.5% (142)	20.8% (104)
Ethnicity/ancestry—% (*n*)		
European (non‐Indigenous North American)	65.4% (599)	61.3% (307)
Non‐European (single or mixed ancestry origin)	17.1% (157)	16.6% (83)
Did not respond	17.5% (160)	22.2% (111)
Prior mental health diagnoses—% (*n*)	68.9% (631)	66.9% (335)
Parent anxiety (baseline)—Mean (SD)	6.83 (5.12)	‐‐
Resilience (baseline)—Mean (SD)	63.90 (9.16)	69.37 (9.72)

*Note*: Parent anxiety is measured by GAD‐7 (Generalized Anxiety Disorder assessment). Resilience is measured by the CYRM‐R (Child & Youth Resilience Measure, Revised) and PMK‐CYRM‐R (Child & Youth Resilience Measure, Revised—Person Most Knowledgeable version). Non‐binary genders are grouped under Other/Did not respond given the small numbers.

Parents who had previously consented to be contacted were sent an invitation email. For children aged 10–18 years old, a separate link was sent to those who were interested in participating. Parents and children completed online surveys via the survey application REDcap (Harris et al., 2009). Participants were sent a link in April 2020 and at regular follow‐up intervals, with questionnaires completed between April 2020 and June 2022. The study was approved by all related institutional research ethics boards. All included participants provided informed consent/assent.

Data were divided into seven time points based on time of collection (Table 2). When two entries were made within one family in the same period (e.g., parents reported on two children), the child with more completed responses was used (*n* = 141). In addition, only participants with data completed on the irritability scale across two or more time points were included.

**TABLE 2:** The measurement timepoints for each measure.

		Irritability	Depression	Anxiety	ADHD	Resilience	Parental anxiety
		TIDES‐6	RCADS‐P	SCARED	SWAN	CTRM‐R/PMK‐CYRM‐R	GAD‐7
T1	4/2020–6/2020	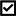				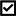	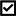
T2	7/2020–8/2020	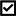					
T3	9/2020–11/2020	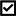					
T4	12/2020–5/2021	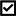					
T5	6/2021‐10/2021	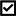					
T6	11/2021–3/2022	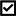					
T7	4/2022‐6/2022	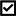	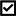	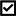	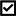		

Abbreviations: CTRM‐R, Child & Youth Resilience Measure, Revised; GAD‐7, Generalized Anxiety Disorder assessment; PMK‐CYRM‐R, Child & Youth Resilience Measure, Revised—Person Most Knowledgeable version; RCADS‐P, Revised Child Anxiety and Depression Scales‐Parent Version; SCARED, Screen for Child Anxiety Related Disorders; SWAN, Strengths and Weaknesses of Attention‐Deficit/Hyperactivity Disorder Symptoms and Normal Behavior Scale; TIDES‐6, The Irritability and Dysregulation of Emotions Scale, 6‐item Short Form.

Data on 1442 children ages 8–18 years were included in the initial sample (response rate 63.5%). Of the 1442 children with data on any outcome measure, completed measures in irritability for at least 2 timepoints were available on 916 unique children. *T*‐test/Chi‐square analyses were conducted to show no significant difference among key demographics (i.e., age group, sex, ethnicity group, family income, and mental health history) between the included and excluded participants for both parent‐report and youth‐report data, ps > 0.05. Data are provided from both parent‐report (*n* = 916) and child/youth‐report (*n* = 501) responses. For the parent‐report sample, 55.5% were boys with mean age of 11.81 (SD = 2.95) years old. For the child‐report sample, 48.9% were boys with mean age of 13.13 (SD = 2.48) years old (see Table 1).

### Measures

#### Demographics

Parent‐report household income, child age/age group, race/ethnicity, gender, and pre‐COVID MH/NDD history were determined using items adapted from the CRISIS questionnaire (Nikolaidis et al., 2021b) an instrument designed by an international collaboration to examine MH during the COVID‐19 pandemic.

#### Questionnaires

Table 2 is the summary of the measure names used and the timing of each measure.

#### Irritability: The Irritability and Dysregulation of Emotions Scale, 6‐item short form (TIDES‐6)

The TIDES‐6 is a 6‐item short version of the TIDES‐13 (Dissanayake et al., 2024), which measures irritability.^1^ Parent‐ and self‐report TIDES‐6 responses were measured across all seven time points of the study.

#### Depressive symptoms: Revised Child Anxiety and Depression Scales‐parent version (RCADS‐P)

Depressive symptoms (children ages 8–18 years) were measured using the Major Depressive Disorder subscale of the parent‐report Revised Child Anxiety and Depression Scales‐Parent Version (RCADS‐P) (Ebesutani et al., 2015; Olatunji et al., 2019). Internal consistencies were found to be high, with Cronbach's alphas ranged from 0.93 to 0.95 for total scores. The 10‐item scale converts to standardized T‐scores, where a T‐score of 65 indicates borderline clinical range (Chorpita et al., 2000).

#### Anxiety symptoms: Screen for Child Anxiety Related Sisorders (SCARED)

Anxiety was measured by parent report (8–18 years) and child report (10–18 years), using the 9‐item Generalized Anxiety Disorder (GAD) subscale of the Screen for Child Anxiety Related Disorders (SCARED) instrument (Birmaher et al., 1997). Cronbach's alphas ranged from 0.74 to 0.93. Scores of nine or greater indicate clinically significant anxiety (Birmaher et al., 1999). T‐scores were used in the model.

#### ADHD symptoms: Strengths and Weaknesses of Attention‐deficit/hyperactivity disorder Symptoms and Normal Behavior Scale (SWAN)

The inattention (9‐item) and hyperactivity/impulsivity (9‐item) subscales of SWAN (Swanson et al., 2012) were used to measure externalizing behaviors. Items were rated on a scale of −3 to +3, where negative values representing strengths/neurotypical behaviors and positive values ADHD symptoms/weaknesses. Cronbach's alphas ranged from 0.86 to 0.96 across raters. Scores for the inattention and hyperactivity/impulsivity subscales were summed separately, with higher total score suggesting greater severity of each type of trait.

#### Resilience: Child & Youth Resilience Measure, Revised (CYRM‐R) and Child & Youth Resilience Measure, Revised—Person Most Knowledgeable version (PMK‐CYRM‐R)

Resilience was measured at T1 (April to June 2020) by parent report (8–18 years) and child report (10–18 years), using the 17‐item CYRM‐R (Jefferies et al., 2019). Items were rated on a scale of one (*not at all*) to five (*a lot*) and assess resilience with unspecified timeframe. Cronbach's alpha was 0.82, suggesting high internal consistency.

#### Anxiety symptoms for parents (and not children): Generalized Anxiety Disorder Assessment (GAD‐7)

Parent anxiety was measured at T1 (April to June 2020) using the 7‐item Generalized Anxiety Disorder assessment (GAD‐7) (Spitzer et al., 2006). Cronbach's alpha was 0.92, suggesting high internal consistency. Increasing scores indicate increasing anxiety.

For all questionnaire measures, scales with missing item inputs were treated as missing data, except for SCARED. For the latter, one item was systematically missing for all participants at one time point. Therefore, the missing data was computed using last observation carried forward, which was empirically tested out to be the most reliable in the context (Cheung et al., 2025).

### Data analysis

Data were analyzed in R (Version 4.4.1) (R Core Team, 2022). First, data patterns, descriptive statistics and pairwise comparisons between adjacent points were carried out. Next, latent growth curve models (LGCM) were used to estimate the irritability trend across the seven time points, assuming a linear trend by parsimony principle. We explored non‐linear models to ensure fit was appropriate. A basic unconditional LGCM was estimated, then a set of conditional models with demographic covariates (i.e., age group, gender, ethnicity, income, mental health history), and psychological covariates (i.e., parental anxiety, resilience) (see Figure 1). The LGCMs were estimated using the RStudio *lavaan* package (Rosseel, 2012), using full information maximum likelihood estimation, which allows missing data. When including exogenous covariates in the conditional models, we specified incomplete data for the exogenous covariates was to be incorporated in the model estimation. The comparative‐fit index (CFI), the Tucker‐Lewis index (TLI), the standardized root mean square residual (SRMR), the root mean square error of approximation (RMSEA), the Akaike Information Criterion (AIC), and the Bayesian Information Criterion (BIC) were used to evaluate model fit indices against rigorous standards (Hu & Bentler, 1999). For better clarity, the parent‐ and self‐report data were presented in two subsections.

To use baseline level and change in irritability over time to predict mental health outcomes, the intercepts and slopes were extracted from the LGCM, and both were used as predictors in a linear regression model to predict internalizing behaviors (anxiety and depression, and externalizing behaviors inattention and hyperactivity/impulsivity).

## RESULTS

### Parent‐report results

Comparison of irritability at adjacent time points revealed a significant increase from TI (the beginning of the pandemic, spring 2020) to T2 (the first summer 2020 after the onset), *t*(661.97) = 2.27, *p* = 0.02, Cohen'*d* = 0.16. Comparison of other adjacent time points were not significant, ps > 0.05.

The overall trajectory was formally tested by the unconstrained latent growth curve model (Unconditional Model; see Table 3 left columns), with *χ* ^2^ = 79.0, *p* < 0.001, CFI = 0.980, TLI = 0.982, SRMR = 0.039, RMSE = 0.052, 90% C.I. [0.39, 0.64], AIC = 26,197, BIC = 26,217 (lower half of Table 4). Overall, the indices suggested satisfactory fit. Both initial irritability (intercept) and change of irritability (slope) were significant, with ps < 0.001. The positive intercept indicated that the irritability was significantly higher at the beginning of the pandemic. The negative slope suggested that over time the irritability level decreased in a linear fashion. The variances of intercept and slope were also significant, ps < 0.001, suggesting individual differences in baseline irritability level and trajectory.

**TABLE 3:** Irritability and mental health outcomes across time points by age, prior mental health history, and gender.

Parent‐report (8–18 years)	*n*	Measure	T1	T2	T3	T4	T5	T6	T7
			Mean (SD)						
Overall	916	TIDES	1.18 (9.05)	2.58 (8.84)	1.53 (8.02)	0.63 (8.50)	0.02 (8.67)	0.07 (8.49)	−0.97 (8.40)
	432	RCADS							56.92 (16.55)
	578	SCARED							7.84 (5.46)
	577	SWAN‐inattn							2.53 (12.57)
	577	SWAN‐hyper							−0.83 (11.80)
Age									
8–12	574	TIDES	1.74 (9.0)	3.85 (8.72)	1.88 (8.05)	0.68 (8.57)	−0.002 (8.61)	−0.01 (8.57)	−1.29 (8.14)
	374	RCADS							56.0 (16.01)
	380	SCARED							7.38 (5.43)
	380	SWAN‐inattn							2.43 (12.20)
	380	SWAN‐hyper							−0.33 (11.99)
13–18	342	TIDES	0.33 (9.07)	1.26 (8.81)	0.95 (7.94)	0.53 (8.39)	0.05 (8.78)	0.22 (8.35)	−0.32 (8.89)
	58	RCADS							62.7 (18.84)
	198	SCARED							8.72 (5.41)
	197	SWAN‐inattn							2.70 (13.30)
	197	SWAN‐hyper							−1.79 (11.41)
Prior MH									
No MH	285	TIDES	−3.32 (8.50)	−1.63 (8.75)	−2.34 (7.23)	−3.06 (8.03)	−4.09 (7.98)	−3.77 (8.13)	−4.52 (7.48)
	179	RCADS							49.3 (13.03)
	204	SCARED							5.04 (4.37)
	204	SWAN‐inattn							−4.56 (10.30)
	204	SWAN‐hyper							−6.00 (10.76)
MH	631	TIDES	3.06 (8.60)	3.73 (8.53)	3.16 (7.78)	2.43 (8.14)	2.00 (8.29)	1.96 (8.02)	1.20 (8.20)
	253	RCADS							62.1 (16.83)
	374	SCARED							9.36 (5.39)
	373	SWAN‐inattn							6.40 (12.01)
	373	SWAN‐hyper							1.99 (11.40)

Child‐report (8–18 years)	*n*	Measure	Mean (SD)	Mean (SD)	Mean (SD)	Mean (SD)	Mean (SD)	Mean (SD)	Mean (SD)
Overall	460	TIDES	−0.03 (9.00)	−0.17 (8.30)	−0.62 (8.09)	−0.10 (8.53)	−0.87 (8.47)	−0.36 (8.34)	−2.41 (8.50)
	329	RCADS							24.01 (11.26)
	329	CES‐DC							9.27 (5.66)
Age									
8–12	281	TIDES	‐‐	‐‐	−1.62 (7.91)	−0.09 (8.37)	−1.51 (8.12)	−1.21 (8.46)	−2.94 (8.55)
	175	CES‐DC							21.6 (10.76)
	175	SCARED							8.07 (5.56)
13–18	220	TIDES	−0.03 (9.00)	−0.17 (8.30)	−0.21 (8.16)	−0.10 (8.67)	−0.22 (8.78)	0.56 (8.13)	−1.77 (8.03)
	154	CES‐DC							26.8 (11.19)
	154	SCARED							10.6 (5.46)
Prior MH									
No MH	166	TIDES	−3.67 (9.43)	−1.04 (9.97)	−3.41 (8.32)	−2.37 (8.46)	−3.98 (7.91)	−2.90 (8.14)	−4.77 (8.69)
	114	CES‐DC							21.32 (11.83)
	114	SCARED							7.65 (6.14)
MH	335	TIDES	0.97 (8.64)	0.09 (7.81)	0.62 (7.70)	1.12 (8.33)	0.72 (8.31)	0.95 (8.15)	−1.05 (8.09)
	215	CES‐DC							25.44 (10.70)
	215	SCARED							10.13 (5.19)
Gender									
Male	245	TIDES	−1.51 (9.59)	−1.94 (8.58)	−1.32 (8.23)	−1.25 (8.18)	−1.05 (8.41)	−1.03 (8.11)	−2.96 (8.59)
	165	CES‐DC							21.42 (10.30)
	165	SCARED							7.98 (5.22)
Female	233	TIDES	1.07 (8.42)	2.20 (7.20)	0.18 (7.87)	1.15 (8.67)	−0.70 (8.59)	0.12 (8.50)	−2.03 (8.43)
	149	CES‐DC							26.71 (11.95)
	149	SCARED							10.56 (5.82)

*Note*: Irritability is measured with TIDES‐6 (The Irritability and Dysregulation of Emotions Scale, brief version). Depression is measured with RCADS (Revised Children's Anxiety and Depression Scale) and CES‐DC (Center for Epidemiologic Studies Depression Scale for Children). Anxiety is measured with Parent and Child versions of SCARED (Screen for Child Anxiety Related Disorders). Inattention and Hyperactivity is measured by the SWAN (Strengths and Weaknesses of Attention‐Deficit/Hyperactivity Disorder Symptoms and Normal Behavior Scale). Valid non‐binary gender data was not reported in the table due to small sample size (self‐report *n* = 15).

Abbreviation: MH, prior mental health history.

**TABLE 4:** Parameters for growth curve models of irritability changes (TIDES) by parent report.

Outcome		Unconditional model				Conditional model with covariates			
Irritability	Covariates	Mean		Variance		Mean/Coefficient		Variance	
		Estimate (S.E.)	*p*	Estimate (S.E.)	*p*	Estimate (S.E.)	*p*	Estimate (S.E.)	*p*
Intercept		**1.37 (0.29)**	<0.001	**57.50 (3.72)**	<0.001	−1.31 (0.83)	n.s.	**49.52 (3.44)**	<0.001
	Age group	n/a				**−2.57 (0.59)**	<0.001		
	MH	n/a				**5.96 (0.63)**	<0.001		
	Gender	n/a				−0.2 (0.57)	n.s.		
	Ethnicity	n/a				−0.53 (0.85)	n.s.		
	Family income	n/a				−0.50 (0.71)	n.s.		
	Parent anxiety	n/a				**0.33 (0.06)**	<0.001		
	Resilience	n/a				**−0.25 (0.04)**	<0.001		
Slope		**−0.23 (0.05)**	<0.001	**0.44 (0.12)**	<0.001	**−0.34 (0.14)**	0.01	**0.43 (0.12)**	<0.001
	Age group	n/a				**0.31 (0.10)**	0.003		
	MH	n/a				−0.02 (0.11)	n.s.		
	Gender	n/a				−0.08 (0.09)	n.s.		
	Ethnicity	n/a				0.14 (0.14)	n.s.		
	Family income	n/a				0.04 (0.12)	n.s.		
	Parent anxiety	n/a				−0.02 (0.01)	n.s.		
	Resilience	n/a				0.004 (0.01)	n.s.		

Model fitness index	Index value	*p*	Index value	*p*	Threshold for good fit
Chi‐square	79.0	<0.001	116.12	<0.001	
CFI	0.980		0.981		>0.95
TLI	0.982		0.978		>0.95
SRMR	0.039		0.026		<0.08
RMSEA [90%CI]	0.052 [0.039–0.064]		0.032 [0.23–0.40]		≤0.05
AIC	26,197		22,873		The smaller the better
BIC	26,217		23,000		The smaller the better

*Note*: Estimates are in unstandardized units. Bolded estimates are statistically significant at *p* < 0.05. Irritability is measured with TIDES‐6 (The Irritability and Dysregulation of Emotions Scale, brief version).

Abbreviations: AIC, Akaike Information Criterion; BIC, Bayesian Information Criterion; CFI, Comparative‐fit index; MH, prior mental health history; RMSEA, root mean square error of approximation; SRMR, Standardized root mean square residual; TLI, Tucker‐Lewis index.

To investigate how demographic and psychological factors may contribute to the trajectory of irritability over time, a Conditional Model with covariates (see Table 4 right columns) was tested, with *χ* ^2^ = 116.12, *p* < 0.001, CFI = 0.981, TLI = 0.978, SRMR = 0.026, RMSE = 0.032, 90% C.I. [0.23, 0.40], AIC = 22,873, BIC = 23,000. The fit indices suggested that the Conditional Model had good fit, and it was similar to the Unconditional Model with *χ* ^2^ = 34.01, *p* = 0.52.

**FIGURE 2:**
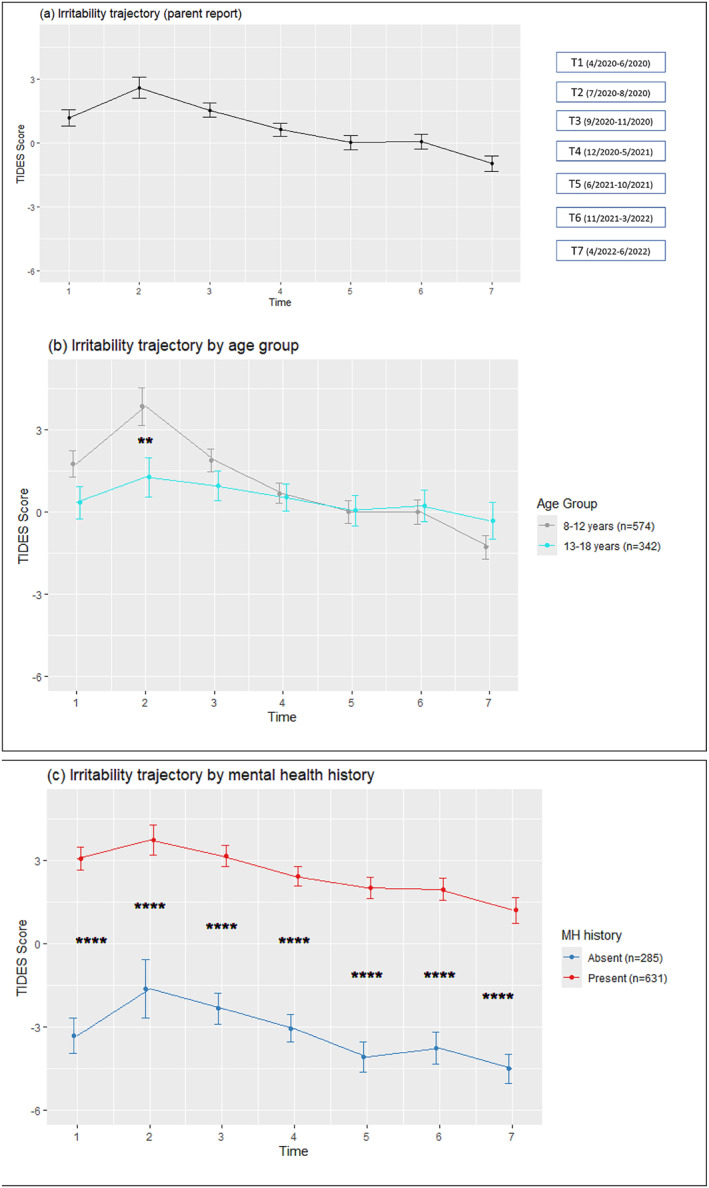
The trajectory of parent‐report irritability over seven time points from April 2020 to Jun 2022. (A) Irritability measured by TIDES‐6. (B) The trajectory of irritability by 8‐12‐year‐old and 13‐18‐year‐old groups. (C) The trajectory of irritability by presence or absence of mental health history including neurodevelopmental conditions and mood and anxiety disorders (differences in all time points were significant, ps < 0.001). Of note, T2 was the first summer after the onset of the pandemic, and T6 covered the time of the Omicron wave. **p < 0.01, ***p < 0.001. Below the graph are indicated the provincial policies on the operation of schools, daycare centers, and recreational center/facilities.

Irritability by age group and MH history across study time points are presented in Table 4. The effect of age on irritability between the two age groups was captured by the slope being significantly associated with by age group (as a covariate), *b* = 0.31, S.E. = 0.10, *p* = 0.003. That is, younger age showed a steeper slope (greater change) over time. Younger age was also associated with higher baseline irritability, with *b* = −2.57, S.E. = 0.59, *p* < 0.001. In a post hoc pairwise analysis, the two age groups were only significantly differed in irritability at T2 (*t*(314.83), *p* = 0.009, *d* = 0.30), with younger children (3.85; SD = 8.72) having higher irritability than older children (1.26; SD = 8.81) (see Figure 2B and Table 3).

Children with mental health history associated with higher intercept (i.e., T1 value) in the Conditional Model, *b* = 5.96, S.E. = 0.63, *p* < 0.001, but not the slope, *b* = −0.02, S.E. = 0.11, *p* = n.s. Children who had mental health history experienced greater irritability symptoms at T1 compared to previously healthy children (3.06 (SD 8.60) versus −3.32 (SD 8.50); *t*(327.97) = 8.27, *p* < 0.001, *d* = 0.75). Their irritability remained higher for the rest of the timepoints (see Figure 2C and Table 3).

Parent anxiety (as measured by GAD‐7) and resilience (as measured by CYRM‐R) were both significant covariates of initial irritability level (i.e., the intercept), *b* = 0.33, S.E. = 0.06, *p* < 0.001, and *b* = −0.25, S.E. = 0.04, *p* < 0.001, respectively. Higher parental anxiety level and lower resilience in children were associated with higher initial irritability level (intercept). However, these two variables were not significantly associated with change over time of irritability (slope).

We investigated the potential impacts of demographic variables in the model. Contrary to expectations, neither gender, ethnicity, nor family income associated with individual differences in the initial irritability level (all predictors of intercept variance ps > 0.05) or individual differences in the change of irritability (all predictors of slope variance ps > 0.05) in the model.

Lastly, we tested initial level of irritability (lower intercept) and change in irritability (more negative slope) predict better mental health outcomes. We used the intercepts and slopes of the models to predict mental health outcomes at the last time point (T7) using linear regression analyses.^2^ Both intercepts and slopes were significant in predicting children's anxiety as measured by SCARED, *F*(2,575) = 54.59, *p* < 0.001, *R* ^2^ = 0.160. In particular, after adjusting for covariates, the change of irritability (slope) was found to be a significant predictor of anxiety level at T7 (April 2022 to June 2022: most pandemic related measures were lifted), with *b* = 1.54, S.E. = 0.56, *p* = 0.006 (see Table S2). Children who experienced decreasing irritability over time had higher levels of anxiety at T7. Similarly, children's depression, as measured by RCADS at T7, was associated with by the slope of the Conditional Model, *F*(2,429) = 64.58, *p* < 0.001. *R* ^2^ = 0.231. Children who experienced decreasing irritability over time had higher levels of depressive symptoms at T7., with *b* = 6.52, S.E. = 1.98, *p* = 0.001. The intercepts and slopes were used to predict inattentive behaviors (inattention subscale of SWAN) at T7, with *F*(2,574) = 76.5, *p* < 0.001. *R* ^2^ = 0.211. Again, children who experienced decreasing irritability over time had higher levels of inattention at T7., with *b* = 3.59, S.E. = 1.25, *p* = 0.004. A similar result was found for hyperactive/impulsive behaviors (hyperactivity/impulsivity subscale of SWAN). The regression was significant, with *F*(2,574) = 72.81, *p* < 0.001. *R* ^2^ = 0.202. Children who experienced decreasing irritability over time had higher levels of hyperactivity/impulsivity at T7, with *b* = 3.56, S.E. = 1.18, *p* = 0.003. For all four regression models, the similar pattern emerged: more negative slopes predict lower symptoms.

We also formally tested out the model fits of both unconditional and conditional models using quadratic modeling. We found that when comparing the unconditional models (no co‐variate was included), the overall model fitness between the linear and quadratic model are highly comparable (see Table S3 for details). We did find a significant quadratic slope (−0.05, *p* = 0.03), confirming the observed non‐linear trend. However, when we moved on to test the conditional models, we found out that while the overall model fitness was again highly comparable between the linear and quadratic models, the quadratic model showed non‐significant linear (−0.45, *p* = n.s.), and quadratic slope (−0.001, *p* = n.s.). In addition, covariates that were found significant (i.e., age group, mental health history, parent anxiety, resilience) in the conditional linear model continued to be significant variables in the conditional quadratic model in predicting the latent intercepts. Age group, unlike in the linear model, was no longer associated with the latent slope (neither linear or quadratic ones, ps > 0.05). Based on this systematic comparison and on parsimony principle, we have weighed the pros and cons and decided to remain our stance to use the simpler model. We aim that would keep the focus back on the key hypothesis, that is to reveal what factors are predicting the changes of irritability over such a turbulent period.

### Self‐report results

**FIGURE 3:**
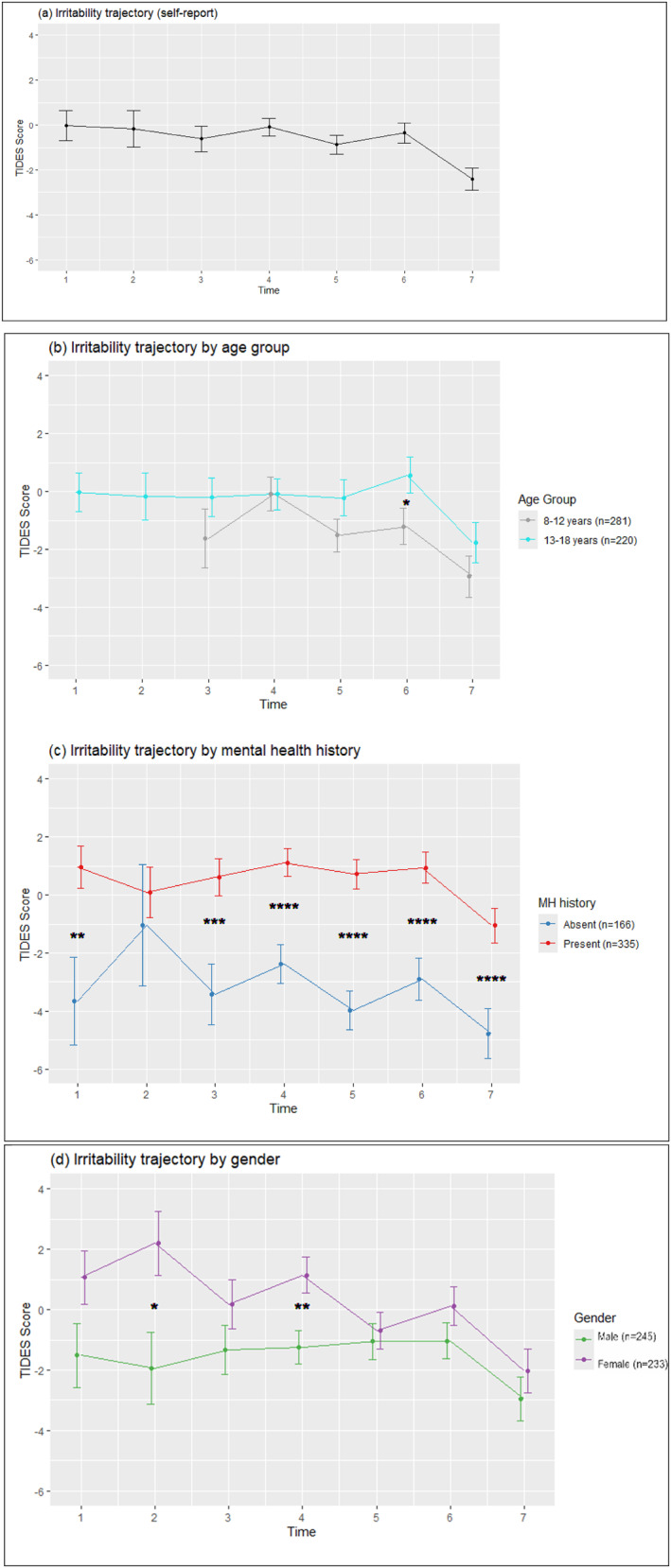
The trajectory of self‐report irritability over seven time points from April 2020 to Jun 2022. (A) Irritability as measured by TIDES‐6. (B) The trajectory of irritability by young and old age groups (8–12 vs. 13–18). The self‐report was introduced for self‐report from T3 onwards. (C) The trajectory of irritability by mental health history. (D) The trajectory of irritability by gender. Non‐binary group was not analyzed and plotted due to small sample size (*n* = 23). Pairwise comparisons were uncorrected: *p < 0.05, **p < 0.01, ***p < 0.005, ****p < 0.001.

While parents reported an initial increase of irritability accompanying a trend of gradual decrease over time in the observed values, the self‐report data showed that irritability was stable over most of the time points except the drop at the end. With pairwise comparisons at each time point, there was no difference in any time points except from T6 to T7, a period that reflected the reopening scheme of the province (see Figure 3A). Children and youth reported a significant decrease in irritability between these two time points, t(614.53) = 3.10, *p* = 0.002.

The overall trajectory was formally tested by the unconstrained LGCM (Unconditional Model; see Table 5 left columns under Model fitness Index), with *χ* ^2^ = 39.61, *p* = 0.017, CFI = 0.982, TLI = 0.983, SRMR = 0.048, RMSE = 0.038, 90% C.I. [0.16, 0.57], AIC = 13,379, BIC = 13,397. Overall, the indices suggested a satisfactory model fit, with a decreasing trajectory of irritability over time, *b* = −0.23, S.E. = 0.09, *p* < 0.001. More importantly, the variances of intercept and slope were significant, ps < 0.001, suggesting individual differences in baseline and trajectory.

**TABLE 5:** Parameters for growth curve models of irritability changes (TIDES) by self report.

Outcome		Unconditional model				Conditional model with covariates			
Irritability	Covariates	Mean		Variance		Mean/Coefficient		Variance	
		Estimate (S.E.)	*p*	Estimate (S.E.)	*p*	Estimate (S.E.)	*p*	Estimate (S.E.)	*p*
Intercept		0.16 (0.47)	n.s.	**61.26 (6.60)**	<0.001	10.99 (7.25)	n.s.	**49.44 (6.95)**	<0.001
	Age group					**−2.35 (1.08)**	0.03		
	MH					**3.27 (1.01)**	0.001		
	Gender					**2.63 (0.92)**	0.004		
	Ethnicity					−0.29 (1.27)	n.s.		
	Family income					−0.72 (1.27)	n.s.		
	Parent anxiety					**0.27 (0.11)**	0.02		
	Resilience					**−0.20 (0.10)**	0.048		
Slope		**−0.23 (0.09)**	**<0.001**	**1.20 (0.34)**	<0.001	−0.87 (1.68)	n.s.	**1.13 (0.31)**	<0.001
	Age group					**0.51 (0.23)**	0.02		
	MH					−0.05 (0.21)	n.s.		
	Gender					−0.12 (0.19)	n.s.		
	Ethnicity					0.10 (0.26)	n.s.		
	Family income					0.16 (0.25)	n.s.		
	Parent anxiety					−0.02 (0.02)	n.s.		
	Resilience					0.004 (0.02)	n.s.		

Model fitness index	Index value	*p*	Index value	*p*	Threshold for good fit
Chi‐square	39.61	0.017	88.37	0.006	
CFI	0.982		0.969		>0.95
TLI	0.983		0.963		>0.95
SRMR	0.048		0.064		<0.08
RMSEA [90% CI]	0.038 [0.016–0.057]		0.023 [0.13–0.32]		≤0.05
AIC	13,379		5894		The smaller the better
BIC	13,391		6021		The smaller the better

*Note*: Estimates are in unstandardized units. Bolded estimates are statistically significant at *p* < 0.05. Irritability is measured with TIDES‐6 (The Irritability and Dysregulation of Emotions Scale, brief version).

Abbreviations: AIC, Akaike Information Criterion; BIC, Bayesian Information Criterion; CFI, Comparative‐fit index; MH, prior mental health history; RMSEA, root mean square error of approximation; SRMR, Standardized root mean square residual; TLI, Tucker‐Lewis index.

To investigate how demographic and psychological factors may contribute to the trajectory of irritability over time, a Conditional LGCM with covariates was tested (Table 5 right columns), with *χ* ^2^ = 88.37, *p* = 0.006, CFI = 0.969, TLI = 0.963, SRMR = 0.064, RMSE = 0.023, 90% C.I. [0.13, 0.32], AIC = 5894, BIC = 6021 (see lower half of Table 5). The fit indices suggested the Conditional Model had better fit than the Unconditional Model (*χ* ^2^ = 58.91, *p* = 0.007).

In self‐reporters, younger age group, presence of mental health history, being a female, higher parent anxiety, and lower resilience were associated with higher initial self‐report irritability (subgroup breakdowns across study time points presented in Table 3; also see Figure 3).

For the age effect, younger age was associated with higher baseline irritability, *b* = −2.35, S.E. = 1.08, *p* = 0.03. This result should be interpreted with caution, however, as the data of the younger group was missing at T1 and T2. For the later time points from T3 onwards, the older (13 to 18‐year‐old group) self‐report higher irritability, compared to the 8 to 12‐year‐old group (see Figure 3B). Only the age difference in T6 was considered statistically significant, however, *t*(360.35) = 2.03, *p* = 0.04, *d* = 0.21. Age group as a covariate to the slope was also found to be significant, *b* = 0.51, S.E. = 0.23, *p* < 0.001, suggesting the older age group had flatter slope.

For the effect of mental health history, its presence was associated with higher intercept (i.e., T1 value) in the Conditional Model, *b* = 3.27, S.E. = 1.01, *p* = 0.001, but not the slope, *b* = −0.05, S.E. = 0.21, *p* = n.s. Children/youth who had mental health history experienced greater irritability symptoms at all time points except T2, ps < 0.01 (see Figure 3C and Table 3).

From the model, females compared to males had higher initial irritability level, *b* = 2.63, S.E. = 0.92, *p* = 0.004. In fact, over subsequent time points, females' irritability remained higher than males although the difference became gradually smaller. At T1, the gender difference was marginally significant, with *t*(162.28) = 1.87, *p* = 0.06, *d* = 0.29 (also see the intercept of the LGCM). The effect was also observed at T2 and T4, with *t*(96.90) = 2.61, *p* = 0.01, *d* = 0.52, and *t*(429.67) = 2.97, *p* = 0.003, *d* = 0.28, respectively. The differences were not statistically significant at subsequent time points, ps > 0.05 (see Figure 3D).

High parent anxiety (as measured by GAD‐7) and low resilience (as measured by CYRM‐R) were associated with higher initial irritability level, with *b* = 0.27, S.E. = 0.11, *p* = 0.02, and *b* = −0.20, S.E. = 0.10, *p* = 0.048, respectively. Consistent with the parent‐report model, it suggested that higher parental anxiety level and lower resilience in children were associated with higher initial irritability level (intercept). Parent anxiety and resilience were not associated with the change of irritability (slope), ps > 0.05.

Ethnicity and family income did not predict initial irritability level (ps > 0.05) or the change of irritability (ps > 0.05) in the model.

## DISCUSSION

The current study investigated the trajectory of irritability among children and youth during the COVID‐19 pandemic. Based on both parent and self‐reports over seven time points from April 2020 to June 2022, irritability initially increased (only in parent‐report data) and then gradually decreased over the course of the changes in public health measures. We discovered that younger age, higher parental anxiety, and having mental health history were significant risk factors in predicting the initial irritability level, while being more resilient was a protective factor. Furthermore, a greater decrease in irritability symptoms over time was associated with lower depression, anxiety, inattention and hyperactivity/impulsivity symptom levels at the conclusion of the study period (i.e., April to June 2022) based on parent reports. As for gender, being female was found to be a significant risk factor *only* in the self‐report model. Ethnicity and family income were not significant in both the models of parent‐ and self‐report irritability.

Our findings about factors that predict irritability during the COVID‐19 pandemic are similar to previous studies looking at factors that were associated with depressive and/or anxiety symptoms. In a systematic review on the mental health impacts of the COVID‐19 panemic on children and youth done by Samji et al. (2022), it was found that older age was associated with worse mental health outcomes. We found an opposite pattern in our parent‐report model, that younger children displayed higher irritability at the acute phase of the pandemic. One may argue that perhaps younger children are more likely to express their distress as irritability, while the older counterpart manifesting it as being depressive or anxious (Stringaris et al., 2012, 2013). It is also possible that parents of younger children were more sensitive in picking up irritability symptoms. When examining the self‐report data, despite the limitation that data of the first two time points were missing, younger children did report a trend of higher irritability level at the intercept.

Those participants who had a positive history of self and family mental history consistently associated with worse initial level and change of irritability. It is consistent with *other* mental health outcomes such as depressive and anxiety symptoms, as found in our cohorts (Cost et al., 2021), and other studies (Madigan et al., 2023; Racine et al., 2020; Samji et al., 2022; Wolf & Schmitz, 2023). It is likely that the high baseline of mood symptoms or other developmental challenges made the individuals more susceptible to the added‐on distress by the pandemic, and that the limited parental support due to parents' own mental health challenges rendered little psychosocial buffer to the children and youth (Korczak et al., 2024; Rizeq et al., 2021). Notably, comparing to the magnitude of the changes in depressive and anxiety symptoms in those reviewed studies, irritability appeared to be a particularly sensitive measure capturing the change of mental health among children.

Gender was not a significant predictor of parent‐report irritability but being female resulted in higher self‐report irritability at the earlier time points during the COVID‐19 pandemic. These results are consistent with a meta‐analysis of depressive symptoms among children and adolescents during the COVID‐19 pandemic (Madigan et al., 2023). The discrepant parent and child/youth reports highlight the importance of sampling responses from both respondents to uncover the full clinical picture. We speculated different effects of gender in parent and self‐respondents may be related to difficulty for parents to accurately identifying internal emotional states of their children. For example, parent‐child agreement between parent‐report versus self‐report anxiety can be very low (Baldwin & Dadds, 2007; Hyland et al., 2022; Olino et al., 2018). In the same meta‐analysis, racial and ethnic‐minority status was not associated with worse depressive symptoms, likely due to the small to slight effect size(Madigan et al., 2023). Thus, we were not too surprised that both ethnicity and family income were not significant predictors in the model.

Our findings also suggest that resilience has been found to be a protective factor against mental illness in face of different life adversities (Galatzer‐Levy et al., 2018). In the context of the pandemic, our results also showed that being resilient buffered effect of irritability. An important implication is that cultivating resilience could be a potential mitigating mechanism to improve mental health outcomes for children and youth (Dvorsky et al., 2021; Yuan, 2021) or adults (Chan et al., 2023; Pearman et al., 2021) who have co‐existing mental health conditions and are at a higher risk for psychopathology. Policy makers could support public health and educational systems to introduce preventive educational programs to foster building resilience. Clinicians in formulating a case conceptualization are also recommended to take into account psychological capital like resilience in their case management (McNicholas & Moore, 2022).

Our findings should be considered in the light of a few limitations. Although we had some attrition of families over the 2‐year period of the study, latent growth curve models we employed allowed for data missingness. However, we cannot completely rule out an alternative explanation that the gradual decrease in irritability was related to a higher attrition rate among those who had higher irritability level (and likely indicating worse mental health conditions). In addition, we did not keep track of the self‐report data at the first two time points, which has limited the interpretation of the intercept and slope of the self‐report models.

Given the richness of the data in such important period of global public health event, other key research questions such as whether a particular public policy such as school closures or lockdowns may have immediate impacts on the psychological well being such as irritability could be answered using the dataset. Public health policy makers could join hands with clinical researchers to capitalize the collected data to develop evidence‐informed policy in the future.

Although the WHO announced the end of the pandemic as a public health emergency on May 4, 2023, the psychological consequences of the pandemic are ongoing and continue to affect a whole generation of children and youth (Grossmann et al., 2021; Taylor, 2022b; The British Academy, 2021). How children and youth are adapting to their post‐pandemic academic and social lives, and how children with mental health and developmental needs are catching up on their training and remediations interrupted by the knockdowns remains to be key public health policy priorities (Dvorsky et al., 2021; McNicholas & Moore, 2022; Rosen et al., 2020; Walker et al., 2020).

In summary, our study highlights the importance of capturing irritability as a mental health outcome in public health crises like the pandemic. Irritability is proven to be a sensitive measure in response to public health events, and a predictor of both internalizing and externalizing symptoms. Adopting a trajectory‐based approach provides more fine‐grained picture on how different demographic and psychological variables may affect the initial level and the change of irritability in face of societal changes. The model also suggests that resilience in children and youth remained an important protective factor in mitigating mental health impact. These findings provide significant policy and clinical implications in understanding important facets of mental health of children and youth in face of the recent global health crisis.

## AUTHOR CONTRIBUTIONS

**Theodore C. K. Cheung**: Conceptualization; formal analysis; methodology; visualization; writing—original draft. **Katherine Cost**: Conceptualization; formal analysis; methodology; supervision; writing—review and editing. **Ronda F. Lo**: Data curation; formal analysis; methodology; visualization; writing—review and editing. **Evdokia Anagnostou**: Funding acquisition; resources; writing—review and editing. **Catherine Birken**: Funding acquisition; resources; writing—review and editing. **Alice Charach**: Funding acquisition; resources; writing—review and editing. **Suneeta Monga**: Funding acquisition; resources; writing—review and editing. **Elizabeth Kelley**: Funding acquisition; resources; writing—review and editing. **Rob Nicolson**: Funding acquisition; resources; writing—review and editing. **Paul Arnold**: Funding acquisition; resources; writing—review and editing. **Christie Burton**: Funding acquisition; resources; writing—review and editing. **Daphne J. Korczak**: Funding acquisition; resources; supervision; writing—review and editing. **Jennifer Crosbie**: Conceptualization; funding acquisition; resources; supervision; writing—review and editing.

## CONFLICT OF INTEREST STATEMENT

Theodore C. K. Cheung has received the Gary Hurvitz Health Scientist Fellowship by the Gary Hurvitz Center for Brain and Mental Health, Hospital for Sick Children, Toronto. Katherine T. Cost has received grants from The Hospital for Sick Children, University of Toronto, and Canadian Institutes of Health Research. They also acted as a statistical consultant for Unity Health and University of Toronto and conference/meeting attendance support from Merit Network. Evdokia Anagnostou has received consultation fees from Roche and Quadrant, research funding from Roche and in‐kind support from Amo Pharma. She has received book royalties from APPI and Springer, and editorial honoraria from Wiley. She holds a patent for the device, “Tully” (formally “Anxiety Meter”) and in‐kind support for all Province of Ontario Neurodevelopmental Network data from the Ontario Brain Institute during the conduct of the study. Catherine S. Birken has received grants from Canadian Institutes of Health Research (CIHR), Heart & Stroke Foundation of Canada, Physician Services Inc, The Edwin S.H. Leong Center for Healthy Children, University of Toronto and Hospital for Sick Children, The Center for Addiction and Mental Health, Walmart Canada Regional Community on addressing food insecurity in children admitted to hospital. Alice Charach has received grants from the Hospital for Sick Children and Leong Center for Healthy Children. J.C. has received grants from CIHR, Ontario Brain Institute, and the Hospital for Sick Children Foundation. Suneeta Monga has received grants from Cundill Center for Youth Depression at the Center for Addiction and Mental Health, book royalties from Springer Publishers, and grants from CIHR outside the submitted work. She has received research support as the holder of the TD Bank Financial Group Chair in Child and Adolescent Psychiatry. Elizabeth Kelley has received grants from Autism Speaks, Neurodevnet, the Bridgewater Foundation, the Masonic Foundation of Ontario, CIHR and the Social Sciences and Humanities Research Council of Canada. Daphne J. Korczak has received grants from CIHR, Hospital for Sick Children, Gary Hurvitz Center for Brain and Mental Health, the University of Toronto, and conference/meeting attendance support from the Canadian Pediatric Society and the Canadian Academy of Child and Adolescent Psychiatry. She has received research support as the SickKids Chair in Child and Youth Medical Psychiatry. The remaining authors have declared that they have no competing or potential conflicts of interest.

## ETHICAL CONSIDERATIONS

The study was approved by the institutional research ethics board at the lead research site (SickKids, 1000070222; 2020‐05‐08) and participating POND sites including Holland‐Bloorview Rehabilitation Hospital (0086; 2020‐05‐06), McMaster Children's Hospital (10,948; 2020‐06‐26), Queen's University (6005107; 2020‐05‐08), and The Lawson Research Institute (115,934; 2020‐06‐05). Informed consent was obtained from all individual participants and/or parents/guardians included in the study.

## Supporting information

Supporting Information S1

## Data Availability

Agree and have included the data availability statement in the manuscript.

## ENDNOTES

^1^
The TIDES‐6 is highly correlated with the TIDES‐13: parent‐report = 0.97; self‐report = 0.96. TIDES‐6 demonstrated excellent internal consistency, with Cronbach alpha of 0.93 in parent‐report, and 0.89 in self‐report data. In term of construct validity, it correlated moderately with measures of ADHD, ODD, and depression scales (*r*s from 0.39 to 0.63 in parent‐report and *r*s from 0.28 to 0.42 in self‐report data).

^2^
Putting all variables in one model resulted in convergence problems. An alternative two‐step approach, with extracted slopes & intercepts in a separate regression model, was taken to address our second hypothesis, that is, to investigate if the initial level and changes in irritability over time might be associated with levels of other mental health symptoms in the later time point (see Table S2).
