# Supplementary material for: Trajectory of irritability in children and youth in Ontario, Canada, throughout the COVID‐19 pandemic
Source: JCPP Adv. 2026 May 6:e70119. Online ahead of print. doi: 10.1002/jcv2.70119 (PMC13339417; doi:10.1002/jcv2.70119)
Supplement: Supplementary file 1 — Supporting Information S1 [file JCV2-9999-e70119-s001.docx]

**Trajectory of Irritability in Children and Youth in Ontario, Canada, throughout the COVID-19 Pandemic**

**Supporting Information**

**Table S1.** COVID-19 Events Timeline in Ontario, Canada during the study timeline (April 2020 to June 2022)

| **Time periods** | **Periods** | **Key events** | **Vaccination events** |
| --- | --- | --- | --- |
| T1 | 4/2020–6/2020 | State of emergency (declared Mar 21, 2020)  1^st^ wave of COVID-19, 1^st^ provincial lockdown, 1^st^ state of emergency declared  Schools closed in person learning, began online learning. Daycares closed.  Recreational centres, parks, playgrounds, retails and restaurants closed. |  |
| T2 | 7/2020–8/2020 | Limited summer camp recreational programs. Most events remain closed. Daycares re-opened. |  |
| T3 | 9/2020–11/2020 | 2^nd^ wave of COVID-19  Primary schools open in person, secondary schools opened hybrid (half in person, half virtual). Daycares opened. |  |
| T4 | 12/2020-5/2021 | 2^nd^ provincial lockdown  Dec 20, 2020: school closed (online learning only)  Jan 21, 2021: 2^nd^ Ontario state of emergency declared  Apr 21, 2021: Delayed March break, 3^rd^ Ontario state of emergency declared  Recreation centres and in-person extracurricular activities closed/cancelled. | Dec 20, 2020: Dose 1 Phase 1 vaccine delivery began (high risk populations)  Mar 21, 2021: Dose 1 Phase 2 vaccine delivery began (key populations, mass delivery)  May 21, 2021: Youth age 12+ became eligible for first dose |
| T5 | 6/2021-10/2021 | School opened hybrid (half in person, half virtual) | Jun 21, 2021: Dose 2 vaccine delivery begins (high risk populations)  Jul 21, 2021: Dose 1 Phase 3 vaccine delivery begins (anyone over 16)  40% of population fully vaccinated in Ontario |
| T6 | 11/2021-3/2022 | Dec 21, 2021: Omicron wave  Jan 22, 2022: School re-opened in person  Mar 22, 2022: Ontario reopening plan began | Nov 21, 2021: Youth age 5-11 became eligible for first dose  Dose 3 vaccine available for eligible populations  75% of population fully vaccinated in Ontario |
| T7 | 4/2022-6/2022 | Jun 22, 2022: Mask mandate lifted | 80% of population fully vaccinated in Ontario  42% of children 5-11 fully vaccinated in Ontario  84% of children 12-17 fully vaccinated in Ontario |

Sources:

AboutKidsHealth. (n.d.). *COVID-19 and kids: Preparing for vaccines*. Retrieved May 15, 2023, from<https://www.aboutkidshealth.ca/article?contentid=4001&language=English>

Canadian Institute for Health Information. (2022, October 13). *Canadian COVID-19 intervention timeline.* Retrieved October 29, 2024, from <https://www.cihi.ca/en/canadian-covid-19-intervention-timeline>

Government of Ontario. (2022, March 9). *Statement from Ontario’s chief medical officer of health*. Retrieved October 29, 2024, from <https://news.ontario.ca/en/statement/1001732/statement-from-ontarios-chief-medical-officer-of-health>

Government of Ontario. (2022, September 12). *COVID-19 vaccines*. Retrieved May 15, 2023, from <https://www.ontario.ca/page/covid-19-vaccines>

Government of Ontario. (2022, September 12). *COVID-19 (coronavirus)*. Retrieved May 15, 2023, from<https://www.ontario.ca/page/covid-19-coronavirus>

Health Infobase. (2024, July 12). *COVID-19 vaccination coverage in Canada*. Retrieved October 29, 2024, from <https://health-infobase.canada.ca/covid-19/vaccination-coverage/>

Ministry of Health. (2022, April 29). *COVID-19 vaccine administration. Version 5.0.* [https://www.health.gov.on.ca/en/pro/programs/publichealth/coronavirus/docs/vaccine/COVID-19_vaccine_administration.pdf](https://can01.safelinks.protection.outlook.com/?url=https%3A%2F%2Fwww.health.gov.on.ca%2Fen%2Fpro%2Fprograms%2Fpublichealth%2Fcoronavirus%2Fdocs%2Fvaccine%2FCOVID-19_vaccine_administration.pdf&data=05%7C02%7Csusan.zahir%40sickkids.ca%7C9f2a64446d624536159d08dcfd11af47%7C3961553ff47e49eb9f6ccf8518914e9a%7C0%7C0%7C638663500514088340%7CUnknown%7CTWFpbGZsb3d8eyJWIjoiMC4wLjAwMDAiLCJQIjoiV2luMzIiLCJBTiI6Ik1haWwiLCJXVCI6Mn0%3D%7C0%7C%7C%7C&sdata=siFvlKDtNUVgkCHAnkqBwL7ytoKTBlC%2FLz%2FdZu0p8FM%3D&reserved=0)

Nasser, S., & Ghania, Y. (2021, August 3). *Ontario releases back-to-school plan with few details for managing COVID-19 cases, outbreaks.* CBC News. Retrieved October 29, 2024, from <https://www.cbc.ca/news/canada/toronto/ontario-back-to-school-covid-19-cases-outbreaks-1.6127752>

Nielson, K. (2020, April 24). *A timeline of COVID-19 in Ontario.* Global News. Retrieved October 29, 2024, from <https://globalnews.ca/news/6859636/ontario-coronavirus-timeline/>

Public Health Agency of Canada. (2022, January 28). *Rapid response: Guidance on the use of booster COVID-19 vaccine doses in adolescents 12 to 17 years of age.* Retrieved October 29, 2024, from <https://www.canada.ca/en/public-health/services/immunization/national-advisory-committee-on-immunization-naci/rapid-response-guidance-use-booster-covid-19-vaccine-doses-adolescents-12-17-years-age.html>

Public Health Agency of Canada. (2024, September 24). *National Advisory Committee on Immunization (NACI): Statements and publications.* Retrieved October 29, 2024, from <https://www.canada.ca/en/public-health/services/immunization/national-advisory-committee-on-immunization-naci.html>

Rodrigues, G. (2021, January 13). *Ontario outlines when people over the age of 80 can be vaccinated for COVID-19.* Global News. Retrieved October 29, 2024, from <https://globalnews.ca/news/7573407/ontario-covid19-vaccinations-plan-seniors/>

Science Table COVID-19 Advisory for Ontario. (n.d.) *Key resources*. Retrieved October 29, 2024, from <https://covid19-sciencetable.ca/key-resources/>

**Table S2.** Irritability pattern associated with mental health outcomes.

| Mental health outcomes | Regression parameters | Slope |
| --- | --- | --- |
| Anxiety (SCARED)​ | F = 54.59, p <.001, R^2^ = .16​ | B_slope_ = 1.54, p = .006​ |
| Depression (RCADS)​ | F = 64.58, p <.001, R^2^ = .23​ | B_slope_ = 6.52, p = .001​ |
| Inattention (SWAN)​ | F = 76.50, p <.001, R^2^ = .21​ | B_slope_ = 3.59, p = .004​ |
| Hyperactivity (SWAN)​ | F = 72.81, p <.001, R^2^ = .20​ | B_slope_ = 3.56, p = .002​ |

Note: The mental health outcomes were measured at Time 7 (T7) when the provincial reopening scheme was implemented. The latent slope of irritability was first exported from the conditioned latent growth curve model, and then it was used as a predictor of the mental health outcomes in four independent regression models.

| Model fitness index | Linear  (unconditional) | | Quadratic  (unconditional) | Linear  (conditional) | Quadratic  (conditional) |
| --- | --- | --- | --- | --- | --- |
| Chi-square |  | 79.0, p<.0001 | 53.80, p<.0001 | 116.12, p<.0001 | 85.12, p=.0001 |
| CFI |  | .980 | .980 | .981 | .988 |
| TLI |  | .982 | .987 | .978 | .982 |
| SRMR |  | .039 | .036 | .026 | .024 |
| RMSEA [90%CI] |  | .052  [.039-.064] | .045  [.031-.059] | .032  [0.23-0.40] | .029  [.019-.038] |
| AIC |  | 26197 | 26180 | 22873 | 22864 |
| BIC |  | 26217 | 26257 | 23000 | 23045 |
| Estimate (S.E.) |  |  |  |  |  |
| Intercept |  | 1.37 (0.29)  p<.0001 | 1.09 (0.32)  p=.0001 | -1.31 (0.83)  p=n.s. | 13.22 (3.54)  p<.0001 |
| (linear) slope |  | -0.23 (0.05)  p<.0001 | 0.06 (0.14)  p=n.s. | -0.34 (0.14)  p=.01 | -0.45 (1.65)  p=n.s. |
| (quad.) slope |  | -- | -0.05 (0.02)  p=.03 | -- | -0.001 (0.24)  p=n.s. |

**Table S3.** Comparison of model fitness with key estimates of linear vs quadratic modeling among unconditional and conditional models
